# Small founding number and low genetic diversity in an introduced species exhibiting limited invasion success (speckled dace, *Rhinichthys osculus*)

**DOI:** 10.1002/ece3.8

**Published:** 2011-09

**Authors:** Andrew P Kinziger, Rodney J Nakamoto, Eric C Anderson, Bret C Harvey

**Affiliations:** 1 Department of Fisheries Biology, Humboldt State UniversityOne Harpst Street, Arcata, CA 95521; 2 U.S.D.A. Forest Service, Pacific Southwest Research Station-Arcata1700 Bayview Drive, Arcata, CA 95521; 3 Fisheries Ecology Division, Southwest Fisheries Science CenterNational Marine Fisheries Service, NOAA, 110 Shaffer Road, Santa Cruz, CA 95060

**Keywords:** Bottleneck, exotic species, founder number, genetic diversity, introduced species, source population, speckled dace (*Rhinichthys osculus*)

## Abstract

Molecular evaluations of successful invaders are common, however studies of introduced species that have had limited invasion success, or have died out completely, are rare. We studied an introduced population of speckled dace (*Rhinichthys osculus*) from northern California, USA that has rapidly increased in abundance but remained restricted to a 25-km stretch of river since its introduction in the mid-1980s. Field and laboratory analyses indicate that invasion success of speckled dace is constrained by the combined effects of multiple predators. The role of bottleneck effects associated with the introduction has not been studied. We assayed variation in seven microsatellite loci and one mitochondrial DNA gene in the introduced population and nine putative source populations to identify the source population and evaluate bottleneck effects. The Trinity River system was supported as the source owing to its genetic similarity and geographic proximity to the introduced population. Consistent with a bottleneck, the introduced population exhibited reduced allelic and haplotype richness in comparison to source populations. Estimates of the genetically effective number of individuals founding the introduced population using nuclear coalescent analyses and a mitochondrial simulation procedure were highly concordant in suggesting that the initial colonizing group was comprised of about 10 individuals. A bottleneck effect in an exotic species exhibiting limited invasion success has rarely been documented and thus introduction of speckled dace represents an important model system for future investigation. Establishing a relationship between genetic diversity and factors limiting invasion success in this system (e.g., predator avoidance) will help determine the extent to which genetic diversity loss has constrained invasion success in speckled dace.

## Introduction

Exotic species are considered second to habitat loss and fragmentation as a threat to global biodiversity (Walker and Steffan 1997; Wilcove et al. 1998). Exotic species can alter natural evolutionary patterns of native species by competitive exclusion, niche displacement, hybridization, introgression, predation, and extinction (Mooney and Cleland 2001). Thus, development of tools for predicting invasion success is important for management of exotic species (Kolar and Lodge 2001).

Genetic diversity is considered essential for establishment and spread of exotic species (Sakai et al. 2001). For pragmatic reasons, genetic diversity is generally evaluated in successful invaders, not in those that have died out or those that failed to expand their range following establishment. However, unsuccessful invaders are key for understanding the importance of genetic diversity for predicting invasion success (Dlugosch and Parker 2008). Unsuccessful invaders are expected to experience dramatic losses in genetic diversity as a result of bottlenecks that reduce adaptive potential and limit spread or cause extinction (Sakai et al. 2001; Reed and Frankham 2003). Thus, to more clearly define the role of genetic diversity for predicting invasion success, studies of introduced species that have had no or limited invasion success are needed to complement studies of successful invaders.

Estimating the number of individuals founding introduced populations using genetic data provides a powerful approach for evaluating bottleneck effects and genetic diversity loss associated with exotic species introductions (e.g., Ross and Shoemaker 2008). Given genotypic data from an introduced population and its source, estimates of founder number can be generated using a coalescent-based maximum likelihood approach (Anderson and Slatkin 2007). Importantly, estimates of founder number can be used to specify the maximum number of alleles per locus that can be retained in an introduced population and apply to all loci in the genome, thereby avoiding issues associated with using a few neutral loci as a proxy for genome-wide patterns. Founder number is also an important demographic parameter for evaluating propagule pressure, which is critical for predicting future establishment and spread of invaders (Lockwood et al. 2005).

We investigated genetic diversity in an introduced population of speckled dace (*Rhinichthys osculus*), a small (usually less than 80 mm standard length) cyprinid fish, which has had limited invasion success since their introduction to the Van Duzen River (northern California, USA) in the mid-1980s (Brown and Moyle 1997; Moyle 2002). Initially suitable habitat conditions and low predation risk allowed establishment and rapid range expansion (Brown and Moyle 1997). However, despite the availability of suitable habitat, speckled dace have remained restricted to a 25-km stretch of the Van Duzen River.

The key trait limiting the invasion success of speckled dace appears to be their inability to evade multiple predators (Harvey et al. 2004). Speckled dace contact sculpins (*Cottus aleuticus and C. asper*) and pikeminnow (*Ptychocheilus grandis*) at their downstream limit in the Van Duzen River. Competition for microhabitat and predation by benthic sculpins, combined with predation by the water-column-occupying pikeminnow, appear to prevent spread of speckled dace in this system (Harvey et al. 2004). However, the severity of the genetic bottleneck resulting from the original introduction event is unknown, thus the extent to which traits associated with predator avoidance may have been lost is unknown. It is possible that dace are within a lag period between establishment and the initiation of range expansion (Sakai et al. 2001) and that evolution of adaptations associated with predator avoidance will allow spread to other portions of the river basin in the future.

We studied invasion genetics of speckled dace by assaying microsatellite and mitochondrial DNA in the introduced population and nine potential source populations. First, we attempted to identify the source population(s) for the introduction. Establishing a source population provides a benchmark to evaluate genetic changes in introduced populations (Dlugosch and Parker 2008). Accuracy of assignment of source populations is directly related to the degree of genetic differentiation among them (Muirhead et al. 2008). Speckled dace exhibit deep mitochondrial DNA divergence among, and sometimes within, major river drainages in their native range (Oakey et al. 2004; Pfrender et al. 2004). Thus, we expected accuracy of assignment of the source population to be at the basin scale or perhaps finer. Second, to gauge the severity of the bottleneck and consequent genetic diversity loss associated with introduction, we used two approaches. First, we estimated the number of founding individuals in the introduced population using our genetic data. Second, we compared levels of genetic diversity in the introduced population to the genetic diversity in likely source populations.

## Materials and Methods

### Sample collection and molecular methods

**Figure 1:**
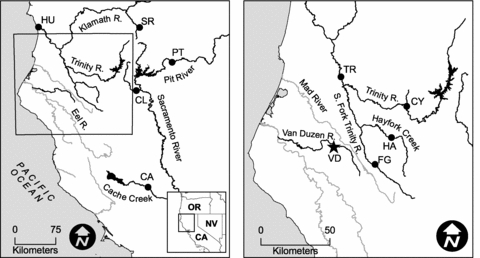
Distribution of sampling sites for speckled dace in northern California, USA. Star indicates the introduced population (Van Duzen River [VD]) and filled circles indicate populations from the native range (Pit River [PT], Clear Creek [CL], Cache Creek [CA], Shasta River [SR], Hunter Creek [HU], Trinity River [TR], Canyon Creek [CY], Hayfork Creek [HA], Forest Glen [FG]). River systems with speckled dace in bold and those that are not known to contain speckled dace in gray.

Speckled dace were collected from the introduced population (Van Duzen River) and nine putative source populations (Fig. 1). Collections included all rivers surrounding the introduced population known to contain speckled dace. The geographic proximity of the sampled source populations makes them reasonable locations for bait collection and live transport to the introduction site. Speckled dace were collected using a seine net or backpack electrofisher, euthanized by administration of an overdose of tricaine methanesulfonate, and preserved in 95% ethanol. Whole genomic DNA was extracted from fin tissue using chelex methods (Miller and Kapuscinski, 1993).

A total of 71 (introduced) and 491 (source) speckled dace were genotyped with seven microsatellite loci (see Table S1). Microsatellite loci amplification was performed using Master Mix (Promega, Madison, WI) in an MJ Research (Waltham, MA) PTC-100 thermal cycler using 10 or 12.5-μl volumes. Polymerase chain reaction (PCR) products were visualized and allele size established using the Beckman–Coulter CEQ 8000 Genetic Analysis System. Allele scores were determined twice and discrepancies were either resolved or no score was assigned.

Tests for conformance to Hardy–Weinberg proportions for each locus in each population were conducted using the Markov chain Monte Carlo approximation of Fisher's exact test implemented in ARLEQUIN 3.1 (Schneider et al. 2000). Loci were tested for null alleles, large allele dropout, and stutter peaks in MICROCHECKER v 2.2.3 (Van Oosterhout et al. 2004). Tests for linkage disequilibrium between all locus pairs in each population were conducted in GENEPOP (Raymond and Rousset 1995) (5000 batches of 2000 iterations). We corrected for multiple tests using Bonferroni methods (Rice 1989).

### Introduction source

Estimates of nuclear genetic differentiation (*F*_ST_) between all population pairs and permutation tests of their significance were implemented in FSTAT V2.9.3.2 (Goudet 1995). PHYLIP (Phylogeny Inference Package, version 3.68) was used to calculate Cavalli-Sforza genetic distance between population pairs and to construct an unrooted neighbor-joining tree (Felsenstein 1993). Branch support was evaluated by conducting a bootstrap analysis from 1000 pseudoreplicates. Multivariate assessment of population differentiation was generated using Discriminant Analysis of Principal Components (DAPC) (Jombart et al. 2010) using the DAPC function implemented in the adegenet package (Jombart 2008) for the R software (R Development Core Team 2009). DAPC performs a preliminary data transformation step using Principal Component Analysis (PCA) to create uncorrelated variables that summarize total variability (e.g., within- and between-group). These variables are used as input to DA, which aims to maximize between-group variability and achieve the best discrimination of genotypes into predefined clusters. The Bayesian clustering algorithm employed in STRUCTURE 2.3.3 (Pritchard et al. 2000; Falush et al. 2003) was used to generate an ad hoc estimate of the most likely number of genetically distinct groups of speckled dace present in the data, and to estimate individual admixture proportions (*Q),* the proportion of each individual's genome assigned to each group. Estimates of the number of genetic clusters present in the data were generated by calculating the log probability of the data (ln Pr[X|K]) and Δ*K* (Evanno et al. 2005) assuming our data consisted of *K*= 1, …, 10 genetically distinct groups (Pritchard et al. 2000). All STRUCTURE analyses were performed using the admixture and the correlated allele frequencies models with no prior population information. The Markov chain Monte Carlo simulation was run for a minimum of 15,000 steps (with 7500 discarded as burn-in) and a minimum of 20 independent runs were conducted at each value of *K*. Inspection of summary statistics (e.g., divergence distances among populations and likelihoods) indicated that run lengths were sufficient for convergence. Graphical depictions of STRUCTURE results were generated using DISTRUCT (Rosenberg 2004).

### Founder number

The effective number of individuals founding the introduced speckled dace population was estimated using maximum likelihood methods implemented in COALIT and NFCONE (Anderson and Slatkin, 2007). Required inputs include nuclear genotypic data from the source and introduced population, number of generations since the introduction event, and demographic history of the introduced population since initial founding (effective carrying capacity and intrinsic rate of increase). While the exact value for most inputs was unknown, we could confidently place limits on the range of possibilities for each parameter. Three separate analyses were conducted, each one assuming a different source population (CY, FG, and HA), from within the Trinity River system, as our findings (see below) indicated that these three locations were the most likely sources. The date of the introduction was set to 10 generations ago. Assuming a 2-year generation time for speckled dace (Moyle 2002), this value would place colony founding approximately 20 years ago, which represents the number of years between the introduction event (mid-1980s; Brown and Moyle 1997) and our field collections in 2004. The current speckled dace population in the Van Duzen River numbers in the thousands (BCH and RJN, personal observation), thus we evaluated a range of effective carrying capacities centered on this observation, including 500, 1000, 2000, 4000, 8000, 16,000. Speckled dace rapidly expanded their range following the original introduction (Brown and Moyle 1997), so we explored intrinsic rates of increase of 0.5, 1.0, 1.5, 2.0, and 3.0. We obtained estimates of founder number using all 90 combinations of effective carrying capacity (500, 1000, 2000, 4000, 8000, 16,000), intrinsic rate of increase (0.5, 1.0, 1.5, 2.0, and 3.0), generations since founding (10), and source population (CY, FG, and HA). Locus CYPG33 was monomorphic in FG and the introduced population and was eliminated from analysis of this source–founder pair because it provided no information for estimating founder number.

### Nuclear genetic diversity

ARLEQUIN 3.1 (Schneider et al. 2000) was used to calculate proportion of polymorphic loci (*P*), allelic richness (*A*), observed heterozygosity (*H*_O_), and Hardy–Weinberg expected heterozygosity (*H*_E_). HP-RARE 1.0 (Kalinowski 2005) was used to calculate standardized private allelic richness (*A*_p_), and standardized allelic richness (*A*_R_), equalized to a sample size of 26 genes using rarefaction. We compared allelic richness, standardized allelic richness, and expected heterozygosity using a two-way analysis of variance (ANOVA) with population and locus considered as random factors. We specifically evaluated the contrast between the introduced population and the nine populations within the species’ native range and a pairwise contrast between the introduced and the most likely source population.

### Mitochondrial DNA

We sequenced a total of 186 individuals from the introduced population and nine putative source populations for a 759-bp fragment of the mitochondrial cytochrome *b* gene. Amplifications were conducted using primers L14724 and H15915 under the following conditions (Irwin et al. 1991): 35 cycles of 94°C for 60 s, 48°C for 60 s, and 72°C for 120 s. Sequences were generated using L14724 at High-Throughput Sequencing Solutions (University of Washington, Department of Genome Sciences). Sequences were aligned in CLUSTALX2 (Larkin et al. 2007). Haplotype frequencies, average number of nucleotide differences between population pairs, haplotype diversity (*h*), and nucleotide diversity (π) were estimated using ARLEQUIN 3.1.

Estimates of the genetically effective number of founders for the introduced population were generated using computer simulations to model the effects of the founding event on haplotype richness. Random samples of founder individuals were drawn (with replacement) from a list of haplotypes and their counts in the source population. A probability distribution was constructed describing the proportion of draws, out of 100,000 trials, which contain two haplotypes, the number detected in the introduced population, assuming the introduced population was founded by 2, …, 20 individuals. The analysis was first run assuming the source was FG (eight haplotypes), the only location containing both haplotypes found in the introduced population. A second analysis was conducted assuming the source consisted of a pooled sample of the three most likely ancestral populations (CY, HA, and FG; 19 haplotypes total), to account for haplotype sampling error. Our analysis follows that of Ross and Shoemaker (2008).

## Results

All nuclear loci were highly polymorphic, ranging from 6 to 48 alleles, with an average of 22 alleles per locus across all populations. On average, <2% of the microsatellite genotypes were missing from the final dataset, and missing data were not characteristic of loci or populations. Of the 70 tests for conformance to Hardy–Weinberg proportions (10 populations at seven loci), five were significant following Bonferroni correction for multiple tests (critical value = 0.0007). MICROCHECKER suggested deviations were primarily due to null alleles; however, no single locus or population consistently departed from expectations, eliminating locus- and population-specific factors as causes for the deviations. A total of two of 210 pairwise tests for linkage disequilibrium were significant following Bonferroni correction for multiple tests (critical value = 0.0002).

### Introduction source

**Figure 2:**
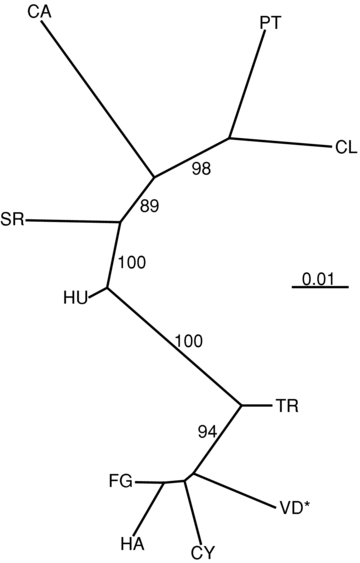
Unrooted neighbor-joining tree generated using PHYLIP. Branch lengths are equivalent to Cavalli-Sforza genetic distance. Bootstrap values are along branches. The introduced population (VD) is indicated by an asterisk.

**Figure 3:**
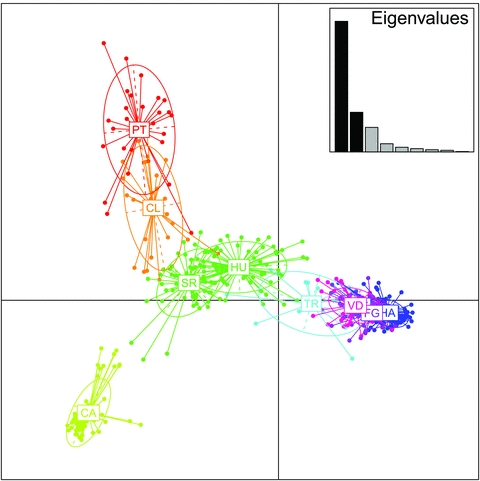
Scatterplot of the first two principal components of DAPC using population locations as prior clusters. Populations are labeled inside their 95% inertia ellipses and dots represent individuals. The inset indicates the eigenvalues of the first nine principal components. Putative source population (CY) is superimposed by the introduced population (VD).

**Figure 4:**
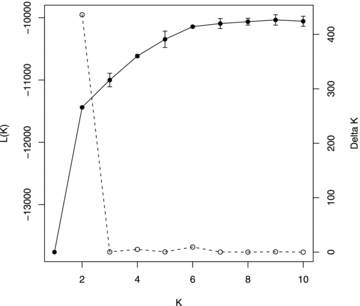
*L*(*K*) (solid line, filled circles, ±SD) and Δ*K* (dotted line, open circles) resulting from 20 runs at each value of 1, …, 10 clusters (*K*).

**Figure 5:**
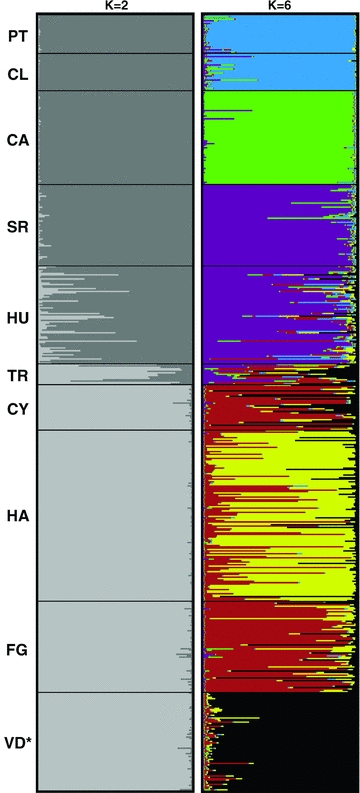
The proportion of each individual's genome (*Q*) assigned to each of two clusters and six clusters inferred by Bayesian cluster analysis with STRUCTURE. The introduced population (VD) is indicated by an asterisk.

Pairwise comparisons of nuclear genetic differentiation (*F*_ST_) indicated that the introduced speckled dace population was most similar to native populations from the Trinity River (TR and CY), 0.0659 and 0.0541, and South Fork Trinity River (HA and FG), 0.1410 and 0.0815, and divergent from all other native populations, 0.2281–0.4807 (Table 1). In the neighbor-joining tree, the introduced population clustered with samples from the Trinity River (CY) and South Fork Trinity River (HA and FG) with 94% bootstrap support (Fig. 2). In the DAPC, 90% of the total genetic variation was captured by the first 45 principal components of PCA and these were used as input to DA. The eigenvalues resulting from DA indicated that the first two axes captured the majority of genetic structure among our speckled dace populations (Fig. 3, inset). The introduced population clustered with samples from the Trinity River (CY) and South Fork Trinity River (FG and HA). In the STRUCTURE analysis, the ad hoc statistic Δ*K* indicated *K*= 2 suggesting our data consisted of two clusters (Fig. 4). One cluster consisted of the introduced population and samples from the Trinity River (CY) and South Fork Trinity River (FG and HA) and the second cluster included samples PT, CL, CA, and SR (Fig. 5). Samples TR and HU contained individuals of mixed ancestry between the two clusters. The probability of the data (*L*[*K*]) arrived at a plateau at *K*= 6, suggesting another lower level of structure featuring six clusters (Fig. 4). In this analysis, the introduced population was assigned to a private cluster. Some degree of admixture of the introduced population with populations TR, CY, HA, and FG is evident from inspection of the individual membership proportions but the 90% probability intervals ranged from 0 to 1, indicating lack of statistical support (Fig. 5).

**Table 1:** Pairwise estimates of genetic differentiation among speckled dace populations. Microsatellite (*F*_ST_) is below diagonal and mitochondrial DNA (average number of nucleotide differences between populations) is above diagonal. Introduced population (VD) is shown in bold

	PT	CL	CA	SR	HU	TR	CY	HA	FG	**VD**
PT	–	2.2	4.9	31.1	27.9	26.2	23.3	23.3	24.1	**23.9**
CL	0.1031	–	4.6	30.8	27.6	26	23.1	23.2	23.9	**23.8**
CA	0.3603	0.2553	–	31.8	29.5	28.3	25.7	25.7	26.5	**26.3**
SR	0.2116	0.1217	0.1939	–	8.2	18.2	25.5	25.6	25.6	**24.6**
HU	0.1796	0.1055	0.1859	0.0438	–	16.6	22.5	22.5	22.6	**21.7**
TR	0.2819	0.2422	0.3588	0.1625	0.0818	–	12.2	12.1	12.4	**11.9**
CY	0.4337	0.4255	0.5228	0.3353	0.2422	0.1007	–	1	1.7	**1.6**
HA	0.4829	0.4773	0.5383	0.3997	0.2972	0.1542	0.1549	–	1.1	**1**
FG	0.3967	0.3837	0.4745	0.3050	0.2092	0.0759	0.0992	0.0745	–	**1.3**
**VD**	**0.4059**	**0.3937**	**0.4807**	**0.3206**	**0.2281**	**0.0659**	**0.0541**	**0.1410**	**0.0815**	–

### Founder number

**Figure 6:**
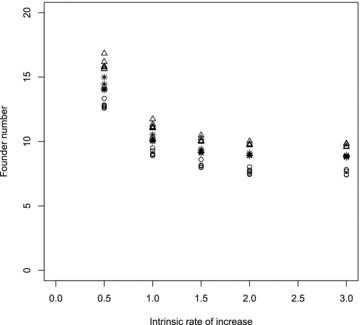
Maximum likelihood estimates of the effective number of founders versus intrinsic rate of increase. Population sources FG (asterisks), HA (circles), and CY (triangles). Results are for generation length of 10 and points are for effective carrying capacity of 500, 1000, 2000, 4000, 8000, and 16,000.

Estimates of the number of founders in the introduced population ranged between 7 and 17 (support limits 6–25), depending on parameter inputs. Intrinsic rate of increase had a modest influence on the founder number estimate. When *r*= 0.5, the estimated number of founders was about 15, but when *r*≥ 1.0, the expected number of founders stabilized at about 10 (Fig. 6). The larger estimated founder number at *r*= 0.5 is attributable to postintroduction drift resulting from smaller population sizes in the initial generations immediately following population founding. Source population and effective carrying capacity had limited influence on founder number estimates, generally causing estimates to vary by less than three individuals when all other parameters were held equal (Fig. 6).

### Genetic diversity

The proportion of polymorphic loci ranged from 0.57 to 1 in the native populations and was 0.86 in the introduced population (Table 2). Standardized private allelic richness ranged from 0.1 to 1.2 in the native populations and was 0.3 in the introduced population. Mean standardized allelic richness ranged from 4.4 to 8.2 in the native populations and was 3.7 in the introduced population. Mean unstandardized allelic richness ranged from 6.0 to 13.0 in the native populations and was 4.7 in the introduced population. Mean observed heterozygosity ranged from 0.34 to 0.61 in the native populations and was 0.47 in the introduced population. The maximum number of alleles observed at a locus in the introduced population was nine.

**Table 2:** Population, sample ID, microsatellite DNA results [sample size (*n*), proportion of polymorphic loci (*P*), number of private alleles (*A*_p_), rarified allelic richness (*A*_R_), allelic richness (*A*), observed heterozygosity (*H*_O_), and expected heterozygosity (*H*_E_)], and mitochondrial DNA results [sample size (*n*), number of haplotypes (*n*_H_), number of private haplotypes (*n*_*p*H_), haplotype diversity (*h*), and nucleotide diversity (π)] in speckled dace. Introduced population (VD) in bold

		Microsatellite DNA							Mitochondrial DNA				
			
Population	ID	*n*	*P*	*A*_p_	*A*_R_	*A* (±SD)	*H*_O_ (±SD)	*H*_E_ (±SD)	*n*	*n*_H_	*n_p_*_H_	*h* (±SD)	π (±SD)
Pit River	PT	28	0.86	0.6	6.2	8.3 (5.0)	0.52 (0.31)	0.54 (0.33)	16	9	6	0.89 (0.06)	0.0029 (0.0019)
Clear Creek	CL	27	0.86	1.0	6.2	8.1 (6.0)	0.56 (0.30)	0.58 (0.30)	24	7	4	0.78 (0.06)	0.0021 (0.0015)
Cache Creek	CA	68	0.57	0.3	4.5	6.7 (7.3)	0.39 (0.38)	0.40 (0.38)	15	4	4	0.64 (0.09)	0.0012 (0.0010)
Shasta River	SR	59	1.00	1.2	7.5	12.4 (8.3)	0.60 (0.26)	0.66 (0.28)	16	9	8	0.82 (0.10)	0.0148 (0.0080)
Hunter Creek	HU	71	0.86	0.6	8.2	13.0 (8.6)	0.61 (0.28)	0.70 (0.32)	16	12	9	0.94 (0.05)	0.0160 (0.0085)
Trinity River	TR	15	0.86	0.4	6.6	7.0 (4.5)	0.60 (0.27)	0.61 (0.29)	15	11	6	0.93 (0.05)	0.0212 (0.0112)
Canyon Creek	CY	33	0.71	0.1	4.5	6.0 (5.3)	0.40 (0.33)	0.39 (0.31)	23	9	7	0.81 (0.07)	0.0029 (0.0019)
Hayfork Creek	HA	124	1.00	0.4	4.4	8.4 (6.7)	0.34 (0.29)	0.37 (0.29)	31	5	3	0.35 (0.11)	0.0015 (0.0011)
Forest Glen	FG	66	0.86	0.3	5.3	8.9 (7.2)	0.44 (0.29)	0.47 (0.32)	15	8	4	0.88 (0.06)	0.0022 (0.0015)
**Van Duzen River**	**VD**	**71**	**0.86**	**0.3**	**3.7**	**4.7 (3.0)**	**0.47 (0.28)**	**0.44 (0.27)**	**15**	**2**	**0**	**0.34 (0.13)**	**0.0005 (0.0005)**

Both allelic richness and standardized allelic richness were lower in the introduced population compared to the nine native populations (*F*= 9.03, df 1,54, *P*= 0.004 for allelic richness; *F*= 10.57, df 1,54, *P*= 0.002 for standardized allelic richness), but expected heterozygosity in the introduced population did not differ detectably from the native populations (*F*= 1.61, df 1,54, *P*= 0.210) (Table 2). A pairwise contrast between the introduced population and the most likely source population (FG) produced a difference in allelic richness (*P*= 0.025), a marginal difference for standardized allelic richness (*P*= 0.085), and no difference for heterozygosity (*P*= 0.748).

### Mitochondrial DNA

**Figure 7:**
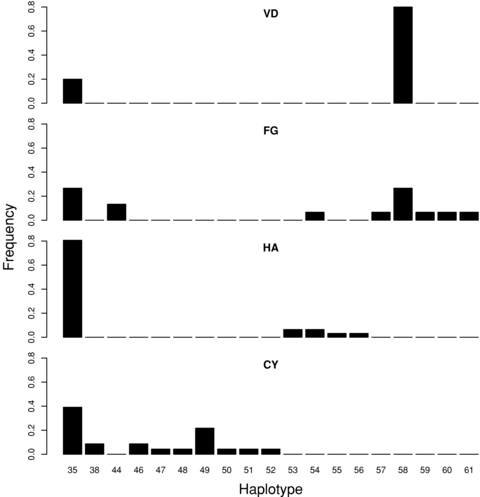
Haplotype frequencies in the introduced population (VD) and the most likely source populations FG, HA, and CY.

A total of 186 mitochondrial DNA sequences were aligned, and 88 variable nucleotide positions defined 61 haplotypes (see Table S2). The introduced speckled dace population exhibited one to two nucleotide differences in comparison to the Trinity River (CY) and samples from the South Fork Trinity River (FG and HA), and marked divergence in comparison to the remaining populations (11.9–26.3 nucleotide differences; Table 1). The introduced speckled dace population contained two haplotypes, one of which was shared only with the South Fork Trinity River sample FG and a second haplotype that was shared with samples from the Trinity River (TR and CY), South Fork Trinity River (HA and FG), and Klamath River (HU) (Fig. 7).

**Figure 8:**
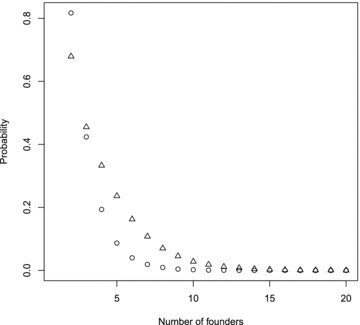
Probability of two haplotypes being represented in different-sized groups of founder individuals. Population source FG only (eight haplotypes, circles) and pooled ancestral source of FG, HA, and CY (19 haplotypes, triangles).

The presence of only two haplotypes in the introduced population contrasted with a range of 4–12 haplotypes present in the native populations (Table 2). The lack of private haplotypes in the introduced population contrasted with three to nine private haplotypes in the native populations. Haplotype diversity was 0.34 in the introduced population and ranged from 0.35 to 0.94 in the native populations. Nucleotide diversity ranged from 0.0015 to 0.0212 in the native populations and was 0.0005 in the introduced population. Ignoring the potential for postintroduction genetic drift, the most likely number of founders bearing only two haplotypes was two regardless of whether the source was assumed to consist of FG only or a pooled sample of the three most likely ancestral populations (CY, HA, and FG) (Fig. 8).

## Discussion

### Introduction source

Our results show the Trinity River system (Trinity River mainstem and its major tributary, the South Fork Trinity River) was the most likely source for the introduced speckled dace population. This result was indicated by parallel trends in estimates of pairwise genetic differentiation, neighbor-joining trees, multivariate plots, and Bayesian clustering analyses. All methods show high levels of microsatellite and mitochondrial similarity between the Trinity River system samples and the introduced population and marked divergence of this founder–source combination from all other populations we examined from the native range. The geographic proximity of the Trinity River system and the introduced population further supports the results of the genetic analyses (Fig. 1).

### Bottleneck

Several lines of evidence support the hypothesis that the introduced population has experienced a severe bottleneck. First, the introduced speckled dace population exhibited divergence from all other populations, including all of the likely source populations, in the analysis of microsatellite variation. Divergence is an indicator of a bottleneck, which causes an increase in frequency of rare alleles among individuals surviving the bottleneck (Dlugosch and Parker 2008). Divergence is expected to be large when the number of individuals surviving the introduction is small. The appreciable divergence we detected between the introduced and source populations in our analyses suggests a severe bottleneck. Second, loss of genetic diversity in the introduced population suggests a bottleneck. Loss of microsatellite allelic richness was most evident and is a strong indicator of population bottlenecks (Allendorf 1986; Spencer et al. 2000). Mitochondrial haplotype loss was even more severe, consistent with the smaller effective population size for mitochondrial DNA in comparison to nuclear DNA (Allendorf and Luikart 2007). In contrast, nuclear heterozygosity was not significantly reduced in the introduced population. This discrepancy likely occurred because heterozygosity estimates derived from microsatellite loci provide almost no signal for detecting bottlenecks unless founder numbers are four or less (Spencer et al. 2000). Further, when founded populations rapidly increase in size, as expected in this case, the effects of drift on heterozygosity are expected to be minimal (Nei et al. 1975).

### Founder number

Over broad ranges of carrying capacity and intrinsic rates of increase, the microsatellite data suggest an effective founder number between 7 and 17. Variation in our estimate could be primarily attributed to the assumed intrinsic rate of increase following introduction (Fig. 6). We hypothesize rapid population growth of speckled dace following introduction for several reasons: (1) field surveys indicate that speckled dace quickly expanded their range in the years immediately following introduction (Brown and Moyle 1997), (2) speckled dace have relatively high fecundities (192–790 eggs per individual; Moyle 2002), (3) speckled dace can be exceptionally good colonizers of disturbed habitats (Pearsons et al. 1992), and (4) the introduced population is currently composed of thousands of speckled dace (BCH and RJN, personal observation). Thus, assuming *r*≥ 1.0 seems reasonable, which suggests a genetically effective founding population of 10. Furthermore, overall genetic drift in a growing, colonized population is influenced most strongly during the early generations when the population is small and there is little density dependence. During such episodes, it is not unrealistic to expect that the variance in reproductive success amongst a small group of founders could be reduced relative to that in later generations. Accordingly, though we have estimated the effective number of founders, this quantity should be close to the census number of (successful) founders.

Computer simulations using mitochondrial DNA haplotypes suggested that the genetically effective number of founders was two. Although smaller than the nuclear-based estimates, mitochondrial DNA is expected to provide an underestimate in comparison to nuclear markers for several reasons. First, due to maternal inheritance, mitochondrial DNA has a smaller effective population size than nuclear DNA. Second, unlike the nuclear DNA simulations, our mitochondrial DNA simulations did not account for loss of haplotypes via drift in the generations immediately following introduction. Lastly, mitochondrial data provide an estimate of the number of female founders, which could be considerably less than the total number of founders. Considering these factors, the nuclear and mitochondrial estimates are highly concordant.

### Single introduction

Our findings are most consistent with a single introduction for multiple reasons. First, our estimate of an actual founding number of 10 individuals is consistent with a single introduction. Second, significant losses of genetic diversity in the introduced population suggest a single introduction, as multiple introductions would likely serve to restore genetic diversity. Lastly, we found no evidence of admixture from multiple founding sources in the Bayesian cluster analysis. Thus, the introduced population of speckled dace in the Van Duzen River apparently contrasts with some successfully introduced populations that have had genetic diversity restored by multiple founding sources and large founder numbers (Stepien et al. 2005; reviewed in Roman and Darling 2007; Brown and Stepien 2009). The absence of repeated introductions despite close geographic proximity of the introduced and native range may be the result of limited fishing pressure and associated bait release combined with regulations that make it illegal to use speckled dace as bait in California.

The mitochondrial DNA analysis points to the South Fork Trinity River site FG as the most likely source, due to one shared haplotype occurring at high frequencies in FG and the introduced population but not found elsewhere (Fig. 7). In contrast, the microsatellite DNA comparisons indicated that the Trinity River sample CY was most genetically similar to the introduced population. However, random divergence between source and founder populations due to drift associated with the introduction event or sampling biases (e.g., Muirhead et al. 2008) could easily mislead source-population assignments at such a fine scale. Divergence between the introduced population and the likely source populations in the microsatellite analysis suggests drift-induced differentiation associated with the original introduction of speckled dace, precluding assignment of a specific Trinity River basin population as the precise source.

### Influence of genetic diversity on ecological performance

The key limitation on the invasion success of speckled dace in the Van Duzen River appears to be their inability to evade multiple predators (Harvey et al. 2004). The severe bottleneck experienced by the introduced population may have caused loss of traits associated with predator avoidance. Previous research has established the heritability of predator avoidance behavior (Endler 1980; Magurran 1990; Reimchen 1994). However, the loss of genetic diversity associated with the introduction may have had no influence on predator avoidance ability. Neutral genetic markers may not accurately reflect variation in traits related to establishment and spread (Allendorf and Luikart 2007; reviewed in Roman and Darling 2007; reviewed in Dlugosch and Parker 2008). Or, it is possible that bottlenecks may facilitate invasion by releasing individuals from former genetic constraints (Carson 1990). Examples of successful exotics founded by very few individuals (e.g., bluegill: Kawamura et al. 2006; fire ants: Ross and Shoemaker 2008; lake trout: Kalinowski et al. 2010) suggest that bottlenecked populations can retain critical behavioral capabilities.

Another possibility is that predator avoidance behaviors may be limited in the introduced population because the source population experiences relatively modest predation risk and, therefore, limited predator avoidance behaviors. The source river lacks pikeminnow and contains few sculpins in much of the river network. Geographic variation in antipredator behavior, according to the presence or absence of major predators in their native range, has been documented for other fish (e.g., three-spined stickleback: Reimchen 1994; Trinidadian guppy: Endler 1980).

Finally, the ecological setting in the Van Duzen River may be such that the spread of speckled dace would be resisted regardless of the relative predator avoidance abilities of the introduced population. An experiment by Harvey et al. (2004) suggested the combination of sculpins and pikeminnow caused a greater negative effect on speckled dace than would be predicted from their separate effects. However, while speckled dace within their native range are susceptible to displacement and consumption by sculpins (Baltz et al. 1982; Moyle 2002), the species does co-occur with sculpins and pikeminnow. Expansion of an introduced population into areas with multiple predators may be a more challenging process than maintaining a population in the presence of those same predators within a species’ native range, where metapopulation processes and, in this example, more complex species assemblages may contribute to persistence.

The relative importance of genetic diversity loss associated with bottlenecking and source population effects could be resolved by conducting laboratory predator avoidance trials. The original multiple predator trials of Harvey et al. (2004) used speckled dace from the introduced population. Comparative analyses that included the introduced and source populations, plus one or more from the native range where both sculpins and pikeminnow occur, could be used to determine the extent to which genetic diversity loss may constrain the introduced population. These experiments will be needed to predict whether the speckled dace populations will evolve characteristics that will allow them to overcome the predation risk environment that appears to be limiting their distribution in the Eel River drainage.

## Data Accessibility

Sample locations, microsatellite data and mitochondrial sequence data deposited at Dryad: doi:10.506/ryad.ht554

## Supporting information

**Table S1.** Microsatellite loci details including allelic richness across all populations, size range (including amplified flanking regions and microsatellite repeats) in base pairs (bp), cycling conditions, and references.

**Table S2.** Mitochondrial DNA haplotype frequencies among speckled dace populations.
