# Supplementary material for: Small founding number and low genetic diversity in an introduced species exhibiting limited invasion success (speckled dace, Rhinichthys osculus)
Source: Ecol Evol. 2011 Sep;1(1):73–84. doi: 10.1002/ece3.8 (PMC3287371; doi:10.1002/ece3.8)
Supplement: Supplementary file 1 [file ece30001-0073-SD1.doc]

**Supplementary Table 1** Microsatellite loci details including allelic richness across all populations, size range (including amplified flanking regions and microsatellite repeats) in base pairs (bp), cycling conditions, and references.

| Locus | Allelic Richness | Size Range (bp) | Cycling Conditions | References |
| --- | --- | --- | --- | --- |
| CYPG3 | 48 | 191-409 | 95^o^C for 60 seconds, 30 cycles of: 95^o^C for 60 seconds, 67^o^C for 45 seconds with -0.5^o^C/cycle, and 72^o^C for 120 seconds. | Baerwald & May 2004 |
| CYPG9 | 8 | 104-128 | 95^o^C for 60 seconds, 30 cycles of: 95^o^C for 60 seconds, 67^o^C for 45 seconds with -0.5^o^C/cycle, and 72^o^C for 120 seconds. | Baerwald & May 2004 |
| LCOL4 | 17 | 227-268 | 94^o^C for 120 seconds, 30 cycles of: 94^o^C for 30 seconds, 60^o^C for 30 seconds, 72^o^C for 30 seconds. | Turner *et al*. 2004 |
| CYPG24 | 25 | 122-194 | 95^o^C for 60 seconds, 30 cycles of: 95^o^C for 60 seconds, 67^o^C for 45 seconds with -0.5^o^C/cycle, and 72^o^C for 120 seconds. | Baerwald & May 2004 |
| RHCA20 | 18 | 94-156 | 92^o^C for 30 seconds, 45 cycles of: 92^o^C for 30 seconds, 50^o^C for 15 seconds, 68^o^C for 5 seconds. Extension at 68^o^C for 120 seconds. | Girard & Angers 2006 |
| CYPG33 | 6 | 83-104 | 95^o^C for 60 seconds, 30 cycles of: 95^o^C for 60 seconds, 67^o^C for 45 seconds with -0.5^o^C/cycle, and 72^o^C for 120 seconds. | Baerwald & May 2004 |
| CYPG27 | 32 | 212-330 | 95^o^C for 60 seconds, 30 cycles of: 95^o^C for 60 seconds, 67^o^C for 45 seconds with -0.5^o^C/cycle, and 72^o^C for 120 seconds. | Baerwald & May 2004 |

**Supplementary Table 2** Mitochondrial DNA haplotype frequencies among speckled dace populations.

| Haplotype | PT | CL | CA | SR | HU | TR | CY | HA | FG | VD |
| --- | --- | --- | --- | --- | --- | --- | --- | --- | --- | --- |
| 1 | 1 |  |  |  |  |  |  |  |  |  |
| 2 | 2 | 5 |  |  |  |  |  |  |  |  |
| 3 | 5 | 4 |  |  |  |  |  |  |  |  |
| 4 | 2 |  |  |  |  |  |  |  |  |  |
| 5 | 1 |  |  |  |  |  |  |  |  |  |
| 6 | 2 |  |  |  |  |  |  |  |  |  |
| 7 | 1 |  |  |  |  |  |  |  |  |  |
| 8 | 1 | 1 |  |  |  |  |  |  |  |  |
| 9 | 1 |  |  |  |  |  |  |  |  |  |
| 10 |  | 2 |  |  |  |  |  |  |  |  |
| 11 |  | 1 |  |  |  |  |  |  |  |  |
| 12 |  | 1 |  |  |  |  |  |  |  |  |
| 13 |  | 1 |  |  |  |  |  |  |  |  |
| 14 |  |  | 8 |  |  |  |  |  |  |  |
| 15 |  |  | 5 |  |  |  |  |  |  |  |
| 16 |  |  | 1 |  |  |  |  |  |  |  |
| 17 |  |  | 1 |  |  |  |  |  |  |  |
| 18 |  |  |  | 7 |  |  |  |  |  |  |
| 19 |  |  |  | 1 |  |  |  |  |  |  |
| 20 |  |  |  | 1 |  |  |  |  |  |  |
| 21 |  |  |  | 1 |  |  |  |  |  |  |
| 22 |  |  |  | 1 |  |  |  |  |  |  |
| 23 |  |  |  | 2 |  |  |  |  |  |  |
| 24 |  |  |  | 1 |  |  |  |  |  |  |
| 25 |  |  |  | 1 |  |  |  |  |  |  |
| 26 |  |  |  | 1 | 1 |  |  |  |  |  |
| 27 |  |  |  |  | 4 | 1 |  |  |  |  |
| 28 |  |  |  |  | 1 |  |  |  |  |  |
| 29 |  |  |  |  | 2 | 1 |  |  |  |  |
| 30 |  |  |  |  | 1 |  |  |  |  |  |
| 31 |  |  |  |  | 1 |  |  |  |  |  |
| 32 |  |  |  |  | 1 |  |  |  |  |  |
| 33 |  |  |  |  | 1 |  |  |  |  |  |
| 34 |  |  |  |  | 1 |  |  |  |  |  |
| 35 |  |  |  |  | 1 | 4 | 9 | 25 | 4 | 3 |
| 36 |  |  |  |  | 1 |  |  |  |  |  |
| 37 |  |  |  |  | 1 |  |  |  |  |  |
| 38 |  |  |  |  |  | 1 | 2 |  |  |  |
| 39 |  |  |  |  |  | 1 |  |  |  |  |
| 40 |  |  |  |  |  | 1 |  |  |  |  |
| 41 |  |  |  |  |  | 1 |  |  |  |  |
| 42 |  |  |  |  |  | 1 |  |  |  |  |
| 43 |  |  |  |  |  | 1 |  |  |  |  |
| 44 |  |  |  |  |  | 2 |  |  | 2 |  |
| 45 |  |  |  |  |  | 1 |  |  |  |  |
| 46 |  |  |  |  |  |  | 2 |  |  |  |
| 47 |  |  |  |  |  |  | 1 |  |  |  |
| 48 |  |  |  |  |  |  | 1 |  |  |  |
| 49 |  |  |  |  |  |  | 5 |  |  |  |
| 50 |  |  |  |  |  |  | 1 |  |  |  |
| 51 |  |  |  |  |  |  | 1 |  |  |  |
| 52 |  |  |  |  |  |  | 1 |  |  |  |
| 53 |  |  |  |  |  |  |  | 2 |  |  |
| 54 |  |  |  |  |  |  |  | 2 | 1 |  |
| 55 |  |  |  |  |  |  |  | 1 |  |  |
| 56 |  |  |  |  |  |  |  | 1 |  |  |
| 57 |  |  |  |  |  |  |  |  | 1 |  |
| 58 |  |  |  |  |  |  |  |  | 4 | 12 |
| 59 |  |  |  |  |  |  |  |  | 1 |  |
| 60 |  |  |  |  |  |  |  |  | 1 |  |
| 61 |  |  |  |  |  |  |  |  | 1 |  |
